# Adaptation of Mycoplasmas to Antimicrobial Agents: *Acholeplasma laidlawii* Extracellular Vesicles Mediate the Export of Ciprofloxacin and a Mutant Gene Related to the Antibiotic Target

**DOI:** 10.1155/2014/150615

**Published:** 2014-01-29

**Authors:** Elena S. Medvedeva, Natalia B. Baranova, Alexey A. Mouzykantov, Tatiana Yu. Grigorieva, Marina N. Davydova, Maxim V. Trushin, Olga A. Chernova, Vladislav M. Chernov

**Affiliations:** ^1^Kazan Institute of Biochemistry and Biophysics, Kazan Scientific Centre of Russian Academy of Sciences, Kazan 420111, Russia; ^2^Kazan Federal University, Kazan 420008, Russia

## Abstract

This study demonstrated that extracellular membrane vesicles are involved with the development of resistance to fluoroquinolones by mycoplasmas (class Mollicutes). This study assessed the differences in susceptibility to ciprofloxacin among strains of *Acholeplasma laidlawii* PG8. The mechanisms of mycoplasma resistance to antibiotics may be associated with a mutation in a gene related to the target of quinolones, which could modulate the vesiculation level. *A. laidlawii* extracellular vesicles mediated the export of the nucleotide sequences of the antibiotic target gene as well as the traffic of ciprofloxacin. These results may facilitate the development of effective approaches to control mycoplasma infections, as well as the contamination of cell cultures and vaccine preparations.

## 1. Introduction

The suppression of mycoplasmas that infect humans, animals, and plants, as well as contaminating cell cultures and vaccines, is a serious problem [1–3]. This is because mycoplasmas rapidly acquire resistance to antibiotics. However, antibiotic therapy remains the primary tool used for the treatment of mycoplasma infections and the decontamination of cell cultures. The most widely used fluoroquinolones, which are synthetic antibacterials, are enrofloxacin, sparfloxacin, ofloxacin, ciprofloxacin, and levofloxacin [3–5]. The mechanisms that facilitate the rapid development of resistance to fluoroquinolones in mycoplasmas remain unclear [6, 7]. The development of resistance to quinolones is associated with mutations in genes that encode antibiotic-targeted proteins, and a limitation is that the antibiotics used to treat microbial cells are not considered to be effective against mycoplasmas [4, 8]. Thus, it would be beneficial to elucidate the mechanisms that facilitate the rapid development of antibiotic resistance to allow the treatment of mycoplasma infections, which appear to be associated with the adaptation of mycoplasmas to stress conditions [9, 10]. The successful implementation of genome projects for a number of mycoplasmas has opened up the possibility of using postgenomic technologies to study their antibiotic resistance processes [11, 12].

A unique species of mycoplasma with adaptive properties is *Acholeplasma laidlawii*, which is a causative agent of phytomycoplasmoses and the main contaminant of cell cultures and vaccines [2, 13, 14]. In our study [9, 10], transcriptome-proteome analysis and nanoscopy were identified as the stress-reactive proteins and genes of *Acholeplasma laidlawii*, which showed that the adaptation of this mycoplasma to stressful factors was related to the production of extracellular vesicles (EVs). The EVs of bacteria are spherical nanostructures surrounded by a membrane (20–200 nm in diameter), which mediate the traffic of a wide variety of compounds that participate in signaling, intercellular interactions, and pathogenesis [15, 16]. Recent studies suggest the possible involvement of EVs in the development of resistance to antibiotics in bacteria [17, 18]. However, there are no previous studies of the roles of EVs in antibiotic resistance in mycoplasmas. Thus, the present study demonstrated the participation of *A. laidlawii* EVs in the development of resistance to fluoroquinolones (ciprofloxacin).

## 2. Materials and Methods

### 2.1. Bacterial Strain and Plasmids

*Acholeplasma laidlawii* PG8 (from the N.F. Gamalei Research Institute of Epidemiology and Microbiology, Moscow, Russia), clinical isolate of *Staphylococcus aureus*, *E. coli* NovaBlue strain, and plasmid vector pGEM-T Easy Vector Systems (Promega) were used in this work.

### 2.2. Cultivation of **A. laidlawii**

*A. laidlawii* PG8 cells were cultivated for 1 day at 37°C in Edward's medium (tryptose, 2% (w/v); NaCl, 0.5% (w/v); KCl, 0.13% (w/v); Tris base, 0.3% (w/v); serum of horse blood, 10% (w/v); fresh yeast extract, 5% (w/v); glucose solution, 1% (w/v); benzylpenicillin (500,000 IE mL^−1^), 0.2% (w/v)) to obtain the control cells. To obtain ciprofloxacin-resistant clones, a mycoplasma culture was grown from a single colony of the laboratory *A. laidlawii* PG8 strain and sequentially inoculated in a broth medium that contained increasing concentrations of the antibiotic. To determine the minimal inhibitory concentration (MIC) of cells, *A. laidlawii* PG8R was subcultured into Edward's medium containing an appropriate concentration of ciprofloxacin. Resistant cultures of *A. laidlawii* PG8R were plated onto solid agar that contained appropriate concentrations of ciprofloxacin and individual colonies were analyzed.

### 2.3. Determination of the MIC

To determine the MIC, the original mycoplasma cell culture was passaged in broth medium with different concentrations of ciprofloxacin: 0.1, 0.2, 0.3, 0.4, 0.5, 0.6, 0.7, 0.8, 0.9, 1.0, and 1.5 *μ*g mL^−1^. The MIC values were determined based on three independent replicates.

### 2.4. Transmission Electron Microscopy and Atomic Force Microscopy

Transmission electron microscopy was performed according to the method of Cole [19]. Samples were fixed with 2.5% glutaraldehyde (“Fluka,” Germany) in 0.1 M phosphate-buffered saline (PBS) (pH 7.2) for 2 h. The fixed samples were then dehydrated using an acetone, ethanol, and propylene series, before postfixing in 0.1% OsO_4_ with 25 mg mL^−1^ of saccharose. After treatment with epoxy resin (“Serva,” Switzerland), ultrathin sections were cut using an LKB-III ultramicrotome (Sweden), which were stained with uranyl acetate for 10 min and lead citrate for 10 min. The stained samples were examined using a JEM-1200EX transmission electron microscope (“Joel,” Japan).

To prepare samples for atomic force microscopy (AFM) analysis, 1 mL aliquots of the *A. laidlawii *PG8 cells and EVs were centrifuged at 12000 rpm for 20 min at room temperature. The pellets were resuspended in 1 mL of PBS × 1 (pH 7.2). The cells were centrifuged once more at 12000 rpm for 15 min at room temperature and repeatedly resuspended in 0.5 mL of the same buffer. The prepared cells were placed onto mica (Advanced Technologies Center, Moscow, Russia) where the upper layer was removed. The cells were air-dried and washed twice with redistilled water. The samples were air-dried after each wash.

AFM imaging was performed using a Solver P47H atomic force microscope (NT-MDT, Moscow, Russia), which operated in the tapping mode with fpN11S cantilevers (*r* ≤ 10 nm, Advanced Technologies Center, Moscow, Russia). The height, Mag (signal from lock-in amplifier), RMS (signal from RMS detector), and phase (signal from the phase detector) were set using the Nova 1.0.26 RC1 software (NT-MDT). The scan rate was 1 Hz and the image resolution was 512 × 512 pixels. Numerical data were expressed as the mean ± SE.

### 2.5. Accumulation of Ciprofloxacin

The presence of antibiotics in the EVs was determined using a fluorimetric method [20, 21]. The fluorescence was measured with a Fluorolog 3 spectrofluorometer (Horiba Jobin Yvon SAS, France) at an excitation wavelength of 282 nm and an emission wavelength of 442 nm. A blank sample that contained an equivalent amount of cells without the addition of ciprofloxacin was used as a control. The results were expressed as nanograms of ciprofloxacin per milligram of protein. All of the experiments were performed at least three times to ensure reproducibility. The mean and standard error were calculated.

### 2.6. Antimicrobial Activity

The Kirby-Bauer disc diffusion method with a ciprofloxacin-sensitive test strain of *Staphylococcus aureus* was used to evaluate the bacteriostatic effects of *A. laidlawii* EVs [22].

### 2.7. Isolation of EVs

EVs were isolated from *A. laidlawii* cultures (logarithmic growth phase) according to the method of Kolling and Matthews, with some modifications [9, 14, 23]. The cells were precipitated by centrifugation at 5000 g for 20 min. The supernatant was filtered through a 0.10 µm filter (Sartorius Minisart, France), and the filtrate was concentrated 20-fold by ultrafiltration (Vivacell 100, 100000 MWCO, “Sartorius Stedim Biotech GmbH”, Germany). Vesicles were collected by ultracentrifugation (100000 g, 1 h, 8°C) with a MLA-80 rotor (Beckman Coulter Optima MAX-E), twice washed, resuspended in buffer (50 mM Tris-HCl, pH = 7, 4; 150 mM NaCl; 2 mM MgCl_2_) and filtered through a sterile filter (Sartorius Minisart, France) with a pore size of 200 nm.

### 2.8. Isolation of DNA from Cells and EVs

DNA was isolated from mycoplasma cells according to the method of Maniatis [24]. DNA was isolated from EVs using a commercial DNA-express kit (“Litekh,” Moscow). Before extracting the nucleic acids, the EV samples were treated with DNAse I and RNase (at 37°C for 30 min).

### 2.9. Sequencing

The PCR primers were constructed by NSF Litekh (Moscow, Russia) using the nucleotide sequences of *A. laidlawii* PG8A genes (GenBank accession number NC_010163): *ftsZ* (Ala1F 5′-ggtttttggatttaacgatg-3′ Ala1R 5′-gcttccgcctcttttattt-3′); spacer 16S-23S of ribosome operon (A16LF 5′-ggaggaaggtggggatgacgtcaa-3′ A23LR 5′-ccttaggagatggtcctcctatcttcaaac-3′); and *parC* (GenBank accession number NC_010163) (AqF15: 5′-ata cgc aat ggg aca aat g-3′; AqR15: 5′-ggt tct tgt tcc tca tca tca-3′). PCR was performed in the following regime: for primers Ala1, 95°C, 3 min (95°C, 20 sec; 52°C, 20 sec; 72°C, 20 sec) (30 cycles); 72°C, 10 min. For primers A23LR, 95°C, 3 min (95°C, 5 sec; 63°C, 5 sec; 72°C, 20 sec) (30 cycles); 72°C, 5 min. For primers Aq15, 95°C, 3 min (95°C, 5 sec; 63°C, 5 sec; 72°C, 5 sec) (35 cycles); 72°C, 5 min.

DNA sequencing was performed using BigDye Terminator v3.1 Cycle Sequencing kits (“Applied Biosystems,” USA) and a 3130 Genetic Analyzer (“Applied Biosystems,” USA). The nucleotide sequences were analyzed using the Sequencing Analysis 5.3.1 program (“Applied Biosystems,” USA) and the NCBI (National Center for Biotechnology Information, http://blast.ncbi.nlm.nih.gov/Blast.cgi) database.

## 3. Results and Discussion

**Figure 1:**
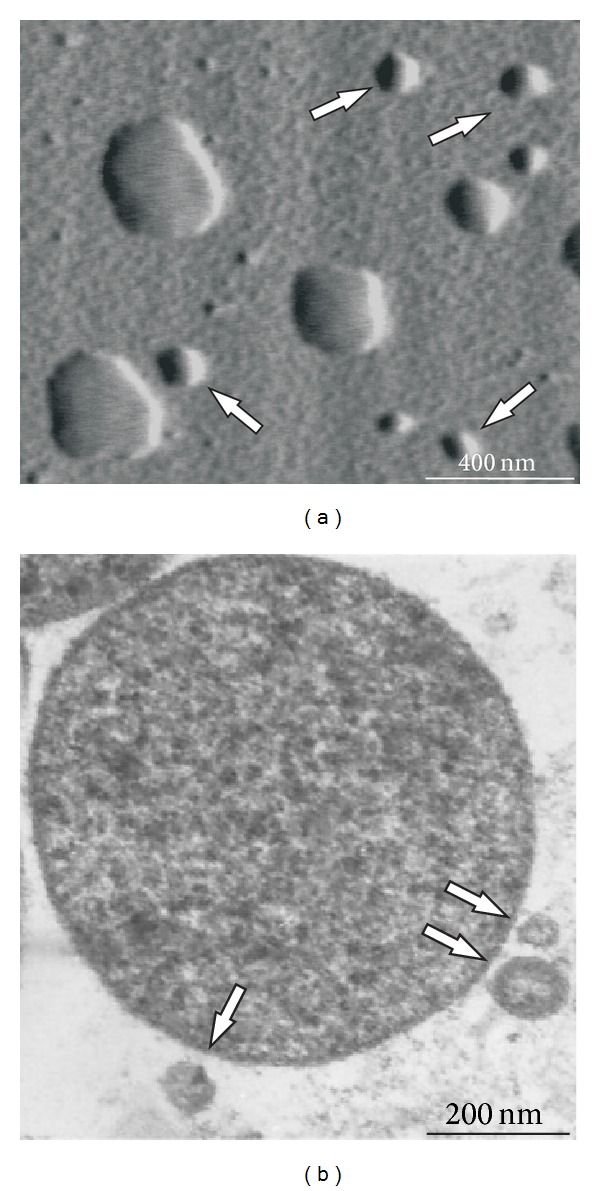
Atomic force microscopy (a) and transmission electron microscopy (b) images of *Acholeplasma laidlawii* PG8 cells and extracellular vesicles (indicated by arrows).

**Figure 2:**
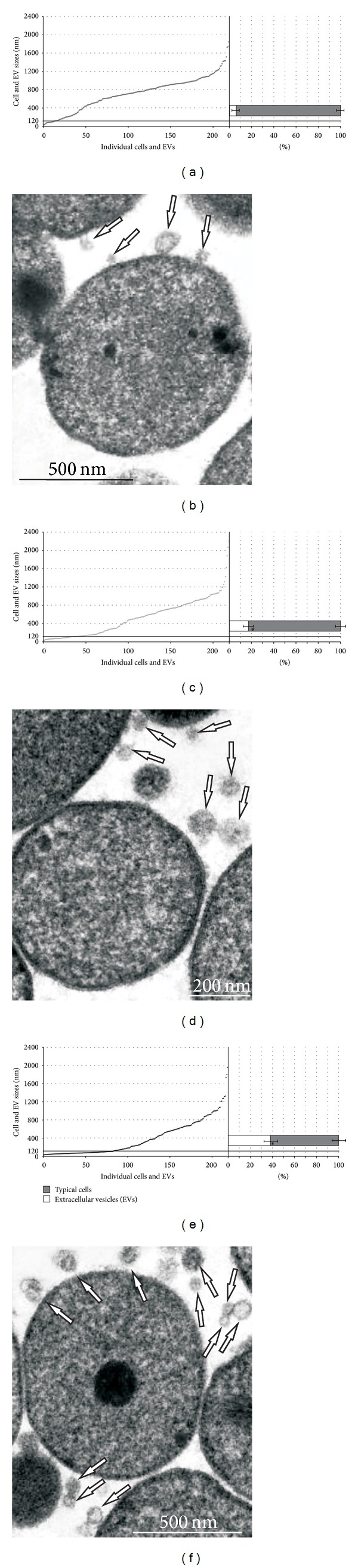
Relationships between typical cells and extracellular vesicles (EVs) (a, c, e) and transmission electron microscopy images (b, d, f) (extracellular vesicles (indicated by arrows)) of *Acholeplasma laidlawii* PG8 without ciprofloxacin (a, b), in the presence of antibiotic **(c, d) and *A. laidlawii* PG8R (e, f) (according to transmission electron microscopy). Each point corresponds to the linear size of individual cells or EVs. *P < 0.05. **Treatment with ciprofloxacin for 80 min.

To determine the role of membrane EVs in the development of resistance to fluoroquinolones in *A. laidlawii*, this study used mycoplasma strains with different levels of susceptibility to ciprofloxacin, that is, PG8 (MIC 0.5 *μ*g/mL) and PG8R (MIC 1 *μ*g/mL). *A. laidlawii* strain PG8R was obtained by stepwise selection from *A. laidlawii* PG8. The analysis of micrographs obtained using variants of transmissive and probe microscopy showed that the mycoplasma cells secreted EVs in normal conditions (Figures 1 and 2(a)). The treatment of the mycoplasma culture with ciprofloxacin elicited a significant increase in the level of vesiculation (sixfold) (Figure 2(b)). The level of vesiculation in* A. laidlawii* PG8R was significantly higher than that in *A. laidlawii *PG8. Recently, similar results were obtained with *Pseudomonas aeruginosa* [25] in another study, which also showed that EVs were involved with the traffic of antibiotics, because the EVs produced by the bacterial cells contained gentamicin [26].

The present study showed that after *A. laidlawii* was cultured in a medium that contained ciprofloxacin, the EVs produced by the mycoplasma contained the antibiotic (0.66 ± 0.15 ng/mg of protein). The EVs that contained ciprofloxacin also exhibited bacteriostatic effects against a clinical isolate of ciprofloxacin*-*sensitive *S. aureus* (Table 1). These results suggest that the EVs of *A. laidlawii* may be involved with the export of ciprofloxacin and possibly with the mechanism of mycoplasma adaptation to antibiotics.

**Figure 3:**
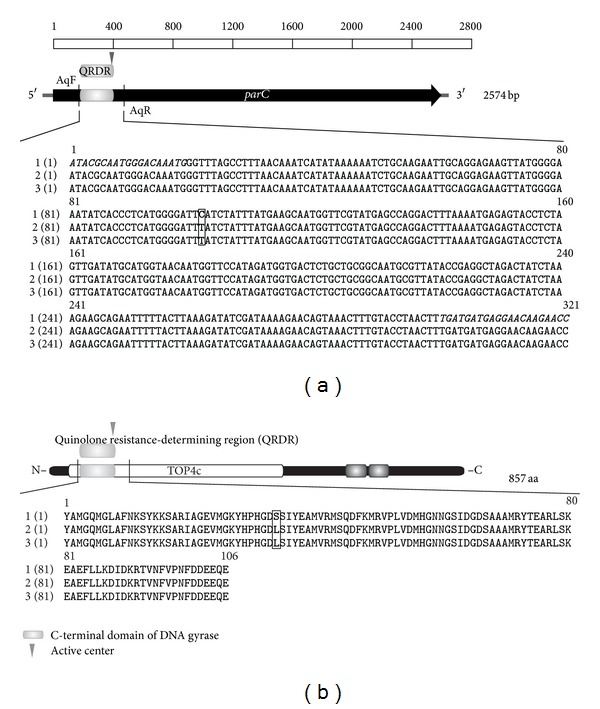
Results of the alignments of the nucleotide (a) and amino acid (b) sequences of the *parC* gene of *Acholeplasma laidlawii* PG8 cells (1), cells of *A. laidlawii* PG8R (2), and the extracellular vesicles of *A. laidlawii* PG8R (3). Italics indicate the sequences of the forward and reverse primers. Nucleotide substitutions and amino acid replacements are enclosed in rectangles, the active site.

The main mechanisms that facilitate the development of resistance to quinolones in bacteria are considered to be associated with mutations in specific loci, that is, the quinolone-resistance-determining regions (QRDRs) of genes that encode antibiotic-targeted proteins [7, 27, 28]. The most significant locus is the QRDR in *parC* of DNA topoisomerase IV [7, 29]. To determine whether this locus was involved with the increased resistance to ciprofloxacin in* A. laidlawii *PG8R, the *parC* QRDRs of the mycoplasma strains were amplified, cloned, and sequenced. The DNA was extracted from mycoplasmas cultivated in broth culture, which were obtained from a single colony grown on solid medium. The DNA sequences isolated from the cells of *A. laidlawii *PG8 and *A. laidlawii *PG8R were not identical (Figure 3(a)). The nucleotide sequence of *parC* QRDR from *A. laidlawii *PG8R contained a C → T transition at position 272, which caused a serine to leucine (Ser (91) Leu) replacement in the amino acid sequence of the antibiotic-targeted protein (Figure 3(b)). Previously, this mutation was found in a strain of *A. laidlawii *PG8B, which was characterized by its elevated resistance to quinolones (6–10 *μ*g/mL) [27]. Thus, the nucleotide sequence of the QRDR in the gene encoding the C subunit of DNA topoisomerase IV in *A. laidlawii *PG8R contained a sense mutation.

The development of resistance to quinolones in bacterial populations associated with point mutations in the QRDR of *parC* should lead to the rapid spread of the corresponding nucleotide sequences in bacterial communities. The main means of spreading antibiotic resistance genes in bacteria is horizontal transfer [30]. Recently, it was shown that the EVs of some bacteria may allow the traffic of genes that encode antibiotic-targeted proteins, thereby mediating the horizontal transfer of nucleotide sequences that determine antibiotic resistance in bacterial populations [31, 32].

Thus, to estimate the potential participation of the mycoplasma EVs in the traffic of the mutant gene that encoded the fluoroquinolone-targeted protein, the EVs of *A. laidlawii *PG8 and *A. laidlawii* PG8R were tested to determine the presence of the nucleotide sequences of *parC* QRDR. Previously [9, 14], we reported that the EVs of *A. laidlawii* PG8 contained the nucleotide sequences of several genes, which can be used as specific markers of the mycoplasma EVs, thereby allowing the detection of EVs and providing a control to detect the absence of bacterial cells in EV preparations. These data were considered when conducting this study.

**Figure 4:**
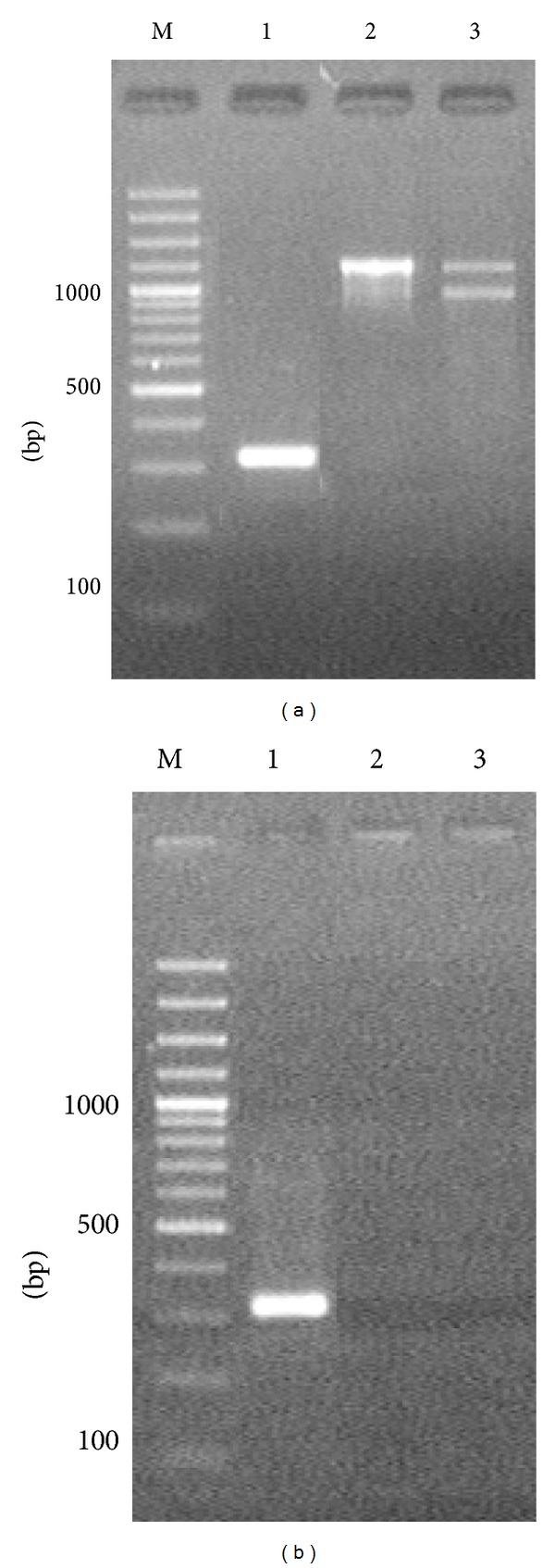
Electrophoregrams of the amplification products of the nucleotide sequences of *parC* (1), *ftsZ* (2), and A23LR (3) of *Acholeplasma laidlawii* PG8, which were obtained by PCR using the total DNA (as a template) isolated from the cells (a) and extracellular vesicles (b) of *A. laidlawii* PG8R. M: molecular weight marker.

PCR using primers that amplified the nucleotide sequences of marker genes in the EVs of *A. laidlawii* PG8 and *parC* QRDR from the mycoplasmas detected the *parC* QRDR-specific PCR signal, where DNA obtained from the EVs of *A. laidlawii *PG8 and *A. laidlawii* PG8R were used as templates (Figure 4). The sequencing data indicated that the PCR signals corresponded to *parC* QRDR from the mycoplasmas. The C → T transition at position 272 was also detected with *A. laidlawii* PG8R (Figure 3). Thus, this study demonstrated that *A. laidlawii* PG8 and *A. laidlawii *PG8R produced EVs that contained *parC* QRDR nucleotide sequences, which were copies of the gene sequences from the respective mycoplasma strains.

*A. laidlawii* PG8R EVs mediated the export of a *parC* QRDR sequence with a C → T transition at position 272, which produced a substitution in the amino acid sequence of the antibiotic-targeted protein. This suggests the possibility that EVs can spread the mutant gene in bacterial communities via horizontal transfer. This capacity was demonstrated recently in *E. coli* and *P. aeruginosa* model systems [33]. A study of similar processes in *A. laidlawii* model systems has yet to be made.

## 4. Conclusions

This study showed that the EVs secreted by *A. laidlawii* cells are involved with the development of the resistance to ciprofloxacin. The results also indicate that the mechanism of antibiotic resistance in this mycoplasma was associated with a mutation in a gene that encoded a quinolone-targeted protein, while the vesiculation level was also modulated. Furthermore, the EVs of *A. laidlawii* mediated the export of the nucleotide sequences of the gene that encoded the ciprofloxacin-targeted protein, as well as the antibiotic. These results demonstrate the necessity of applying appropriate approaches to the development of effective methods for controlling mycoplasma infections, as well as contamination of cell cultures and vaccines.

## Figures and Tables

**Table 1:** Ciprofloxacin content and bacteriostatic activities of the extracellular vesicles of *Acholeplasma laidlawii* PG8R.

Test samples	Ciprofloxacin (5 g/mL)	Extracellular vesicles of *A. laidlawii* PG8R*	
		Native	Destroyed
Ciprofloxacin content, ng/mg of protein	—	—	0.66 ± 0.15
Lysis area (mm)**	21.2 ± 0.3	3.4 ± 0.21 (*P* < 0.05)	8.8 ± 0.16 (*P* < 0.05)

*Mean ± standard deviation.

**Native extracellular vesicles of *A. laidlawii *PG8R were used as a control to estimate the bacteriostatic effects of the vesicle components.
